# Vegetable-Enriched Brownies: A Healthier Twist on a Classic Treat

**DOI:** 10.3390/nu17010184

**Published:** 2025-01-03

**Authors:** Katarzyna Petka, Kinga Topolska

**Affiliations:** Department of Plant Products Technology and Nutrition Hygiene, Faculty of Food Technology, University of Agriculture in Krakow, 21 Mickiewicz Av., 31-120 Krakow, Poland

**Keywords:** brownie, fat replacers, plant-based modification

## Abstract

Background/Objectives: In response to concerns about high-fat and low-fiber diets, this study modified a traditional brownie recipe by replacing butter with plant-based ingredients, including sweet potatoes, red beans, beetroot, zucchini, pumpkin, lentils, and spinach. The goal was to increase vegetable consumption while identifying the best vegetable fat replacer using sensory and instrumental analyses. Methods: Chemical analyses were conducted to measure dry matter, protein, fat, ash, and dietary fiber, alongside texture, color, and sensory evaluations. Results: The butter replacement led to a significant reduction in fat content, up to three-fold, and a decrease in caloric value by 57% while increasing dietary fiber and ash levels. Sweet potato brownies (SPB) had the highest fiber and ash content, while zucchini brownies (ZB) exhibited improved texture due to greater moisture retention. Sensory assessments showed that classic brownies (CB) ranked highest in appearance, taste, and texture, while lentil (LB) and spinach brownies (SB) had lower acceptability. Conclusions: Incorporating plant-based ingredients into brownies significantly improves their nutritional profile but can affect sensory attributes. Further optimization is needed to balance nutritional benefits with consumer preferences, offering a promising approach to increasing vegetable intake through a popular dessert.

## 1. Introduction

Brownie is a kind of chocolate cake made up of wheat flour, eggs, fat, sugar, and chocolate as the basic ingredients. Its texture can either be cakey or fudgy, depending on the individual’s preferences [1]. It is popular among consumers because of its attractive color, extraordinary aroma, and sweet taste [2]. Brownie is usually consumed as dessert; however, the mixture of ingredients mentioned above makes this popular cake packed with high energy value but low in dietary fiber [3]. It is popular among consumers because of its attractive color, extraordinary aroma, sweet taste, and not-too-fluffy texture [2].

The frequent, excessive consumption of products abundant in sugar and fat could result in an increased risk of non-communicable diseases (NCDs). Meanwhile, the burden of NCDs, such as obesity, type 2 diabetes, cardiovascular problems, etc., has rapidly increased worldwide, making them a leading cause of disability, morbidity, and mortality [1,4]. According to the World Health Organization [5], these diseases accounted for about 41 million deaths globally.

Among diet-related risk factors, not only sugar consumption but also the amount of fat (with special attention to saturated fatty acids) is of importance. In this context, the brownie recipe is the main concern of nutritionists and also consumers. The fact is that—independently of the delicious, sweet taste of this popular cake—the nutritional quality of brownies strongly needs improvement. This is why it is interesting to look for alternative raw materials to substitute partially or replace the whole amount of “problematic,” from a dietetic point of view, ingredient [4,5].

Until now, numerous attempts have been made and described in the literature to enhance the nutritional value of brownies. Most of these efforts have focused on the substitution of wheat flour. Additionally, several studies have investigated the use of fat replacers, such as banana puree and black bean puree, as exemplified by Wei and Fleischer [6,7].

The choice of plant-derived products, namely vegetables, was due to the fact that they play a crucial role in human nutrition [8]. Regular consumption of a diet rich in vegetables has undeniable beneficial effects on health. Vegetables in the daily diet have been strongly associated with overall good health and reduced risk for some types of cancer, gastric ulcers, as well as heart disease, diabetes, and other chronic diseases [8].

The choice of plant-derived products, namely vegetables, stems from their crucial role in human nutrition [8]. Regular consumption of a diet rich in vegetables has undeniable beneficial effects on health. Vegetables in the daily diet are strongly associated with overall good health, including a reduced risk of certain cancers, gastric ulcers, heart disease, diabetes, and other chronic diseases [8]. Incorporating vegetables into cake recipes to create healthier variants of popular desserts aligns with consumer needs and the current recommendations of nutritionists. 

In this context, our study aimed to investigate the potential of several vegetables to enhance the nutritional profile of brownies. Vegetables such as zucchini, spinach, sweet potato, lentils, red kidney beans, pumpkin, and beetroot offer diverse health benefits that can enrich the cake recipe while taming their presence for consumer acceptability. Moreover, considering their structure and texture, these vegetables blend easily into a puree, making them particularly suitable for integration with the other brownie ingredients. Zucchini, for instance, is low in calories (14 kcal/100 g) and high in water (96.5%), making it ideal for hydration and low-calorie diets. It is rich in vitamin C and potassium, with mucilage providing digestive benefits, particularly for gastrointestinal disorders [9,10]. Spinach delivers a dense nutritional profile, including flavonoids, carotenoids, and vitamins A, C, and E, which provide antioxidant benefits. It is high in dietary fiber and minerals like iron (34% RDI/100 g), calcium, and magnesium, supporting cholesterol control, weight management, and bone health [11,12]. Sweet potato is rich in beta-carotene, a precursor to vitamin A, which supports vision and immune health. It also provides dietary fiber, vitamins C and B6, potassium, and magnesium, promoting cardiovascular and metabolic health. Its low glycemic index and antioxidants further enhance its role in disease prevention [13,14]. Lentils are a protein-rich, high-fiber food that supports gut health and glycemic control. They are packed with iron, zinc, and folate, making them ideal for preventing nutrient deficiencies. Polyphenols in lentils provide antioxidant and anti-inflammatory benefits [15]. Red kidney beans are a source of protein, fiber, and key micronutrients like iron and potassium, supporting heart and metabolic health. Their low-fat content and bioactive compounds, including polyphenols, reduce cholesterol and inflammation, lowering the risk of chronic diseases [16,17]. Pumpkin is nutrient-dense and high in beta-carotene, promoting antioxidant and immune functions. It is rich in dietary fiber, while its seeds contain protein, unsaturated fats, and essential minerals. Polyphenols enhance their anti-inflammatory and anticancer properties [18,19]. Beetroot is notable for its nitrates and betalains, which support cardiovascular health and reduce inflammation. It also contains polyphenols, fiber, and minerals that aid metabolic health and weight management. Additionally, beetroot is prominent in sports nutrition for improving endurance [20,21,22].

Our goal was to use them in the brownie recipe, so they give the possibility of introducing more vegetables into the diet so as to tame their presence a bit. Additionally, all the proposed cake variants are very easy to make, which can also be an additional advantage for consumers and encouragement for further vegetable modifications. The point is, however, to find the best vegetable fat replacer using both sensory attributes and instrumental methods.

## 2. Materials and Methods

### 2.1. Characteristics of Raw Materials

Medium-sized chicken eggs (approximately 55 g each), dark chocolate (70% cocoa), wheat flour (type 550), and white sugar were purchased from a local supermarket. For the classic brownie (CB), high-fat butter with 82% content, and for modification: pumpkin (*Cucurbita pepo*), red beet (*Beta vulgaris*), baby spinach (*Spinacia oleracea*), red lentils (*Lens culinaris*), zucchini (*Cucurbita pepo*), sweet potato (*Ipomoea batatas*), and red kidney beans (*Phaseolus vulgaris*). The eggs, butter, and chocolate were refrigerated, while the sugar and flour were stored in a dry room at a temperature of 15–20 ± 1 °C with a relative humidity of less than 60%. All raw plant materials were stored under refrigerated conditions at 4 ± 1 °C. The detailed list of ingredients used for brownie production is presented in Table 1:

### 2.2. Plant-Based Puree Preparation

The preparation of plant-based fat substitutes for the cake-baking process was carefully designed to ensure consistent quality and optimal integration into the batter.

The pumpkin, red beet, zucchini, and sweet potato were washed, peeled, steamed at 100 °C for 20 min, and then blended into smooth purees using a high-speed blender (Kenwood Blend-X Pro BLM800, Havant, UK, 2018). The blending process ensured a lump-free consistency, essential for seamless incorporation into the brownie batter. Spinach was rinsed, steamed at 100 °C for 10 min, and blended similarly, while red lentils were rinsed, boiled at 100 °C for 15 min, and blended into a uniform puree. Red kidney beans were drained, rinsed thoroughly to remove any residual salt or preservatives, and blended until smooth. All vegetable pulps were cooled to approximately 45 ± 1 °C using a blast chiller (Redfox, Prague, Czech Republic, 2010) immediately after preparation and were used promptly in the brownie production.

### 2.3. Cakes Preparation

**Scheme 1:**
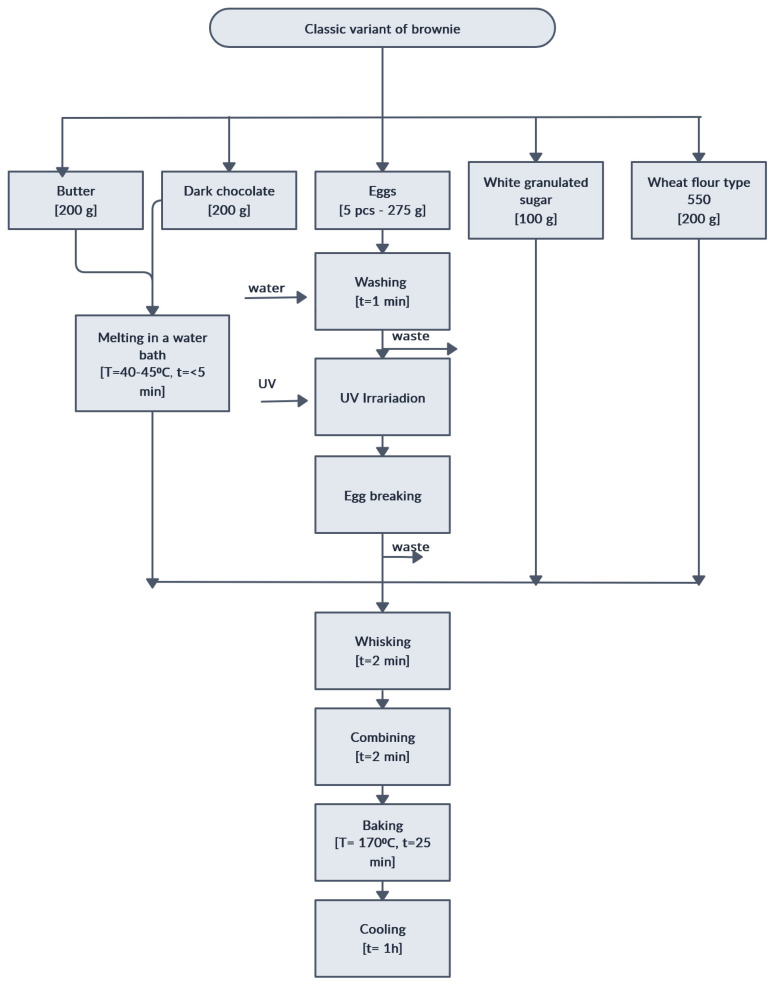
Flowchart of the classic brownie production process.

The classic variant of the brownie was prepared according to the technological procedure outlined below in Scheme 1:

Eggs were washed, subjected to UV irradiation, and weighed. White granulated sugar was then added. Butter and dark chocolate were melted together in a water bath at 96–98 °C for approximately 5 min. Wheat flour (type 550) was incorporated into the mixture, which was whisked for about 2 min. All ingredients were then combined and mixed for an additional 2 min. The batter was baked at 170 °C for approximately 25 min using silicone baking molds (24 × 24 cm) in a convection-steam oven (Retigo Orange Vision, Retigo, Rožnov pod Radhoštěm, Czech Republic). After baking, the brownie was allowed to cool naturally for about 1 h. 

**Scheme 2:**
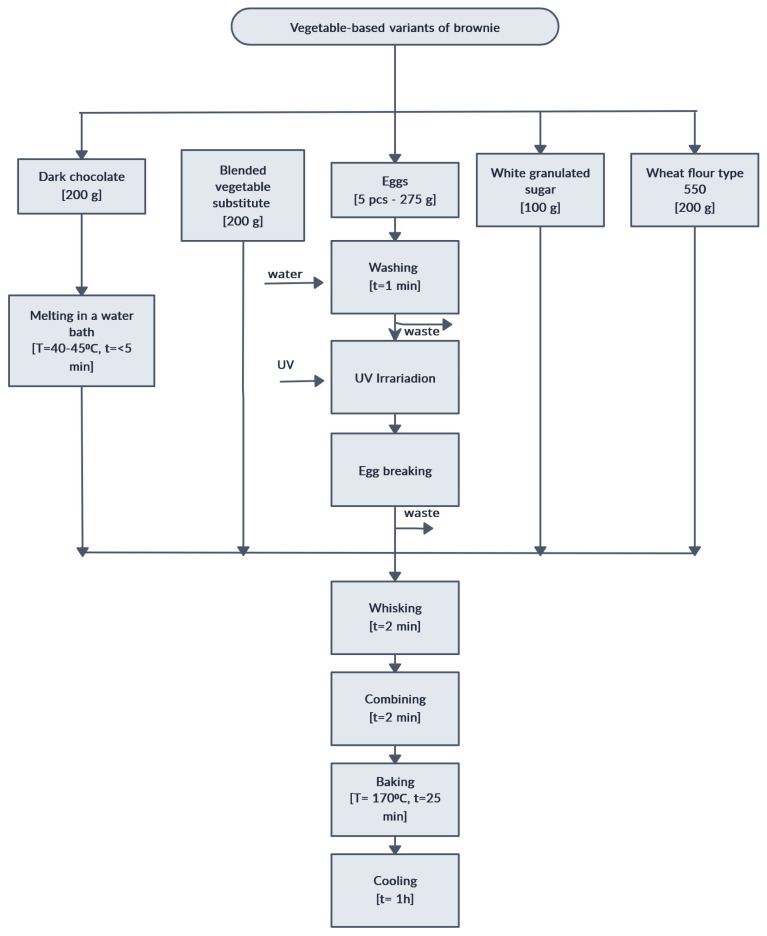
Flowchart of the vegetable-based brownie production process.

The vegetable-based variants were prepared following the same procedure; however, butter was replaced with the corresponding blended vegetable substitute (Scheme 2).

All variants of the brownies, including the classic version and the vegetable-based one, were prepared in three separate batches. Sensory evaluation was conducted after the cakes were baked and cooled, while samples designated for chemical analyses were finely ground, sealed in airtight plastic containers, and stored at −20 °C. All chemical analyses were performed in triplicate.

**Figure 1:**
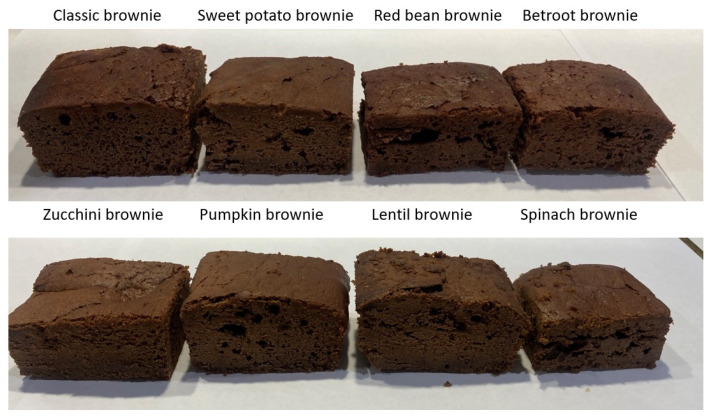
All variants of baked and chilled brownies.

The picture (Figure 1) shows all the variants of the baked brownie, after they have cooled at room temperature. The samples prepared in this way formed the basis for organoleptic evaluation.

### 2.4. Chemical Analysis

The dry matter content was assessed using the dry-weighing technique, as outlined in the PN-ISO 712:2002 [23] standard, employing a Venticell 55Plus dryer (BMT, Brno-střed, Czech Republic). Protein quantification followed the Dumas method per the PN-EN ISO 16634-2:2016 [24] standard, utilizing a TruSpec N device (LECO, Dallas, TX, USA) with a nitrogen-to-protein conversion factor of 6.25. Ash content was measured following PN-EN ISO 2171:2010 [25], using a Nabertherm muffle furnace (LE6/11/B150, Lilienthal, Germany). Dietary fiber was determined using the enzymatic–gravimetric approach described by AOAC 991.43 [26], with a Megazyme kit and verification via the TDF Controls KIT (Megazyme, Lansing, MI, USA). Fat content was established using an in-house validated protocol, where supercritical CO_2_ extraction was carried out using a Leco TFE 2000 analyzer. In brief, 1 g of sample and 1 cc of 2-propanol were weighed into diatomite-filled thimbles, sealed, and analyzed at 100 °C and 9000 psi (LOQ = 0.1 g/100 g fresh mass). The energy value of the cakes was determined using the Atwater factors: 4 kcal/g for carbohydrates and protein, 9 kcal/g for fat, and 2 kcal/g for fiber, as per Kunachowicz et al. [27]. The formula is expressed as follows: E = (P × 4) + (F × 9) + (C × 4), where E represents the total energy content in kilocalories (kcal), P is the amount of protein in grams, F is the amount of fat in grams, and C is the amount of carbohydrates in grams. This calculation is based on the standard Atwater factors, which assign 4 kcal per gram of protein, 9 kcal per gram of fat, and 4 kcal per gram of carbohydrates. 

### 2.5. Instrumental Analysis

#### 2.5.1. Texture Analysis

The evaluation was carried out using a TA.XT Plus texture analyzer (Stable Micro Systems, Godalming, UK). The Texture Profile Analysis (TPA) test involved compressing a square cake sample, measuring 4 × 4 cm, twice using a cylindrical probe with a 100 mm diameter (SMS P/100). The sample was compressed at a speed of 2 mm/s to achieve 60% deformation, with a 5-s pause between the first and second compressions. The resulting force–time curves were analyzed using the Texture Exponent software (v. 2.0.7.0, Stable Micro Systems, UK), which enabled the determination of several key textural parameters. Hardness was measured as the maximum force (in Newtons) required to reach the set deformation of the product, corresponding to the peak of the first compression. Adhesiveness was defined as the work (in N·mm) performed by the probe during the upward movement required to overcome the forces of attraction between the sample and the probe, calculated as the area under the force curve below the *x*-axis. Springiness was determined as the ratio of the time taken between the probe’s contact with the sample and the end of its downward motion in the second and first compressions. Cohesiveness was calculated as the ratio of the areas under the curves of the second and first compressions, while chewiness (in Newtons) was determined as the product of hardness, cohesiveness, and elasticity.

#### 2.5.2. Color Evaluation

The color of the brownies was assessed following the method described by Flores [28] using a CM-3500d spectrophotometer from Konica Minolta in the CIE system (Konica Minolta, Inc., Osaka, Japan). Measurements were taken by placing the device’s measuring head at the center of each brownie. Color values were recorded on the CIE L∗ a∗ b∗ scale, with three measurements taken per sample, and the average values were reported. L∗ represented lightness (ranging from 0 = black to 100 = white), a∗ indicated redness (+a∗) or greenness (−a∗), and b∗ reflected yellowness (+b∗) or blueness (−b∗). Additionally, the total color difference (ΔE) between the vegetable-enriched cakes and the classic brownie was calculated using the formula ΔE = √[(ΔL*)2 + (Δa*)2 + (Δb*)2], where ΔL*, Δa*, and Δb* represent the differences in the respective color coordinates.

### 2.6. Sensory Evaluation

The sensory evaluation was conducted using a panel of 20 assessors and a two-stage evaluation method. First, a 5-point descriptive rating scale was employed to assess specific attributes such as appearance, color, texture, flavor, and overall acceptability in accordance with the PN-EN ISO 5492:2009 standard [29]. Subsequently, a hedonic test was conducted to evaluate the overall preference for brownies using a 9-point hedonic scale, where 1 represented “extremely dislike” and 9 represented “extremely like,” following the guidelines established by Lyon et al. [30]. Panelists were provided with four randomly selected samples, each measuring 1 cm × 1 cm × 1 cm, taken from the center of the brownies, which had been chilled immediately after baking. Additionally, the acceptability index was determined in accordance with the methodology outlined by Fernandes and Salas-Mellado [31] using the corresponding formula.

$$LA\;\left(\%\right)=\frac{Score\times 100}{9}$$

### 2.7. Statistical Analysis

Analysis of variance (ANOVA) was performed using Statistica ver. 13.3 (Tibco Software Inc., Palo Alto, CA, USA) to assess the effect of the differentiating factor—the presence or absence of vegetable modification—on instrumental and chemical parameters. Post hoc comparisons were carried out using Duncan’s test, with statistical significance set at *p* < 0.05. Means were calculated from three independent replicates unless otherwise specified. Correlation analyses were also conducted to examine relationships between sensory attributes and instrumental parameters, providing additional insights into the influence of measured properties on sensory perceptions, as shown in the table in Section 3.5. The correlations were calculated using Pearson’s correlation coefficient (r), which measures the strength and direction of the linear relationship between two variables. This analysis was performed using the statistical software Statistica 13.3 (Tibco Software Inc., Palo Alto, CA, USA). Prior to performing the correlation analysis, the data were checked for normality using the Shapiro–Wilk test, ensuring that the assumptions for Pearson’s correlation were met.

## 3. Results

### 3.1. The Effect of Recipe Modification on Brownie Chemical Composition

Table 2 shows the results of the analyses of moisture, protein, fat, dietary fiber, and ash contents, as well as the calculated carbohydrate level of the cakes (the classic variant and its vegetable modifications) examined.

As seen in Table 2 for moisture content, the classic brownie exhibited the lowest value of this parameter, and the differences between CB and all vegetable-enriched samples were statistically significant. Beetroot brownie was characterized by over twice as much moisture as CB (*p* < 0.05).

In terms of protein content, the classic brownie had a significantly lower value than all the cake variants except for SB. The average level of this macronutrient in vegetable-enriched brownies was 7.48–8.39%, with the highest values obtained in lentil brownies (over 12% higher protein content than in CB), followed by red bean brownies with the statistically highest (ca. 10% higher, as compared to the classic version). As expected, fat content was notably highest in the classic brownie (butter as a fat source). When vegetables were used instead of fat, the values of this parameter decreased (*p* < 0.05), with the lowest results gained for pumpkin and spinach cakes (ca. 29.5% of CB fat content).

The fiber content was significantly different among the brownie variants containing plant-derived raw materials, ranging from the lowest value (5.80 ± 0.68 g/100 g) observed for SB to the highest values for RBB and SPB (7.95 ± 0.12 g/100 g and 8.15 ± 0.05 g/100 g, respectively).

Significant differences were also observed in ash content among cake samples, where the classic brownie had the lowest value. Three vegetable-enriched variants were characterized by significantly enhanced levels: those with pumpkin—by 31%, with red beans—34%, and sweet potato—36%. Lastly, the total carbohydrate content varied significantly among the brownies and ranged from 30.50 ± 0.31 g/100 g for BB and 39.95 ± 0.10 g/100 g for ZC. 

**Figure 2:**
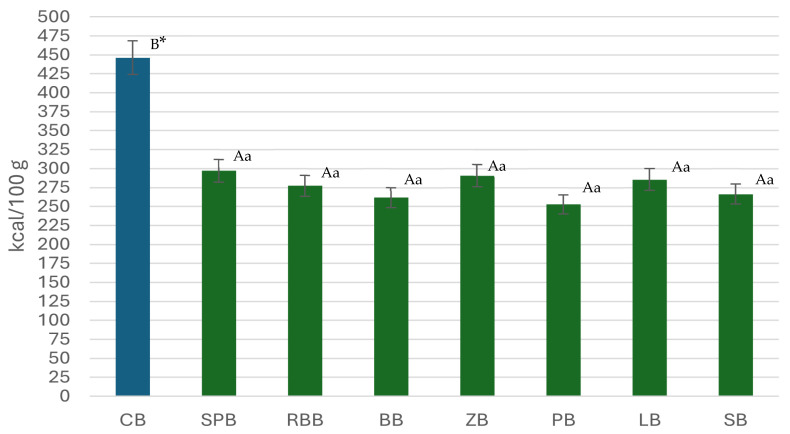
Caloric contribution of brownie variants. CB—classic brownie, SPB—sweet potato brownie, RBB—red bean brownie, BB—beetroot brownie, ZB—zucchini brownie, PB—pumpkin brownie, LB—lentil brownie, SB—spinach brownie. * A, B—different letters denote statistically significant differences between the mean results for the classic brownie and the modified variants (*p* < 0.05); a—different letters denote statistically significant differences between the modified brownie variants (in dependence on the vegetable additive used) (*p* < 0.05).

On the basis of the determinations made, the energy value of the cakes was calculated using the appropriate formula [9]. It was shown that the highest energy value was obtained by classic brownie (446.09 ± 25 kcal/100 g). Vegetable variants ranged from 252.65 ± 8 kcal/100 g for pumpkin cake to 296.94 ± 15 kcal/100 g for sweet potato brownie (Figure 2). 

### 3.2. Texture Analysis

**Figure 3:**
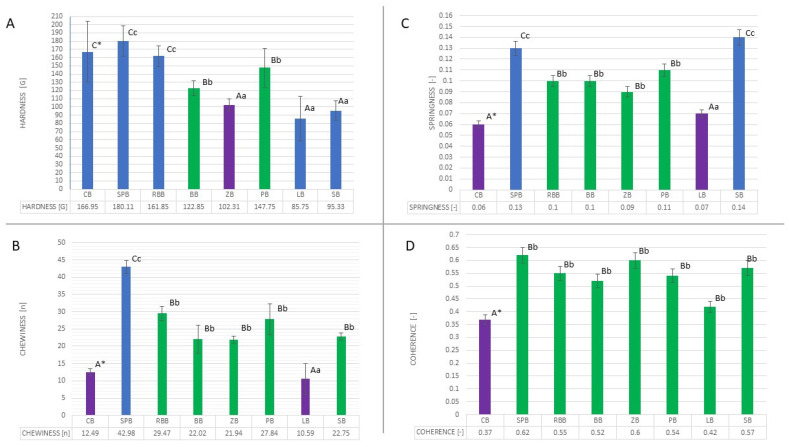
Texture analysis of brownie variants: (**A**) hardness, (**B**) chewiness, (**C**) springiness and (**D**) coherence. CB—classic brownie, SPB—sweet potato brownie, RBB—red bean brownie, BB—beetroot brownie, ZB—zucchini brownie, PB—pumpkin brownie, LB—lentil brownie, SB—spinach brownie. * A, B, C—different letters denote statistically significant differences between the mean results for the classic brownie and the modified variants (*p* < 0.05); a, b, c—different letters denote statistically significant differences between the modified brownie variants (in dependence on the vegetable additive used) (*p* < 0.05).

In Figure 3, the results obtained from the texture analysis are presented in detail, highlighting the differences between the classic brownie (CB) and the vegetable-based variants.

For hardness (Figure 3A), the classic brownie (CB) exhibited a value of 166.95 g, representing a baseline for comparison. Sweet potato brownie (SPB) and red bean brownie (RBB) demonstrated similar or slightly higher hardness values (180.11 g and 161.85 g, respectively), with no statistically significant differences observed compared to CB (*p* > 0.05). However, beetroot brownie (BB) and pumpkin brownie (PB) were significantly softer, with values of 122.85 g and 147.75 g, respectively (*p* < 0.05). Among the softest were zucchini brownie (ZB), lentil brownie (LB), and spinach brownie (SB), with hardness values of 102.31 g, 85.75 g, and 95.33 g, respectively, all significantly different from CB (*p* < 0.05). These findings suggest that the incorporation of certain vegetable purees can substantially reduce the hardness of brownies.

For chewiness (Figure 3B), CB had the lowest value of 12.49, serving as a reference. In contrast, SPB was significantly chewier, with a value of 42.98 (*p* < 0.05), which could be attributed to the structural properties imparted by sweet potato puree. Other variants, such as RBB, BB, ZB, and PB, demonstrated moderate chewiness, ranging from 21.94 to 29.47, all significantly higher than CB but lower than SPB (*p* < 0.05). Interestingly, LB displayed the lowest chewiness value of 10.59 among the modified variants, which did not differ significantly from CB (*p* > 0.05).

Regarding coherence (Figure 3C), CB exhibited the lowest value at 0.37, indicating weaker internal binding compared to the other brownies. All vegetable-based variants, including SPB, RBB, BB, ZB, PB, LB, and SB, displayed significantly higher coherence values, ranging from 0.42 to 0.62 (*p* < 0.05). These results highlight the potential of vegetable purees to enhance the structural integrity of the brownies, which may influence their sensory perception and handling characteristics.

For springiness (Figure 3D), CB and LB had the lowest values (0.06 and 0.07, respectively), indicating limited elasticity. In contrast, SPB and SB exhibited the highest springiness values (0.13 and 0.14, *p* < 0.05), suggesting a more elastic texture likely influenced by the composition of the vegetable purees used. RBB, BB, ZB, and PB displayed moderate springiness, with values ranging from 0.09 to 0.11, all significantly higher than CB but lower than SPB and SB (*p* < 0.05).

### 3.3. Color Evaluation of Brownies

Table 3 shows the results of the color analysis of the brownies (the classic variant and its vegetable modifications). The cakes were named by their abbreviations: CB—classic brownie, SPB—sweet potato brownie, RBB—red bean brownie, BB—beetroot brownie, ZB—zucchini brownie, PB—pumpkin brownie, LB—lentil brownie, SB—spinach brownie. The control brownie (CB) had relatively higher lightness (L*) values (26.33 ± 0.7) compared to other variants. Sweet potato brownie (SPB) and lentil brownie (LB) exhibited the highest lightness values (30.0 ± 2.75 and 30.2 ± 2.82, respectively), both showing significantly lighter colors than the rest of the samples. In terms of redness (a*), significant variations were observed across the samples. Spinach brownie (SB) had the lowest a* value (6.97 ± 0.29), indicating a reduced red hue, while red bean brownie (RBB) and beetroot brownie (BB) had significantly higher a* values (14.1 ± 0.55 and 14.36 ± 0.15, respectively), contributing to their more intense red color. Yellowness (b*) also varied significantly, with SPB and LB exhibiting the highest values (11.38 ± 1.01 and 12.12 ± 0.17, respectively), while SB had the lowest (5.69 ± 0.38), indicating a reduced yellow hue in spinach-enriched brownies. The total color difference (ΔE) was significantly higher in zucchini brownie (ZB) (2.37 ± 1.16) and spinach brownie (SB) (2.05 ± 1.12), indicating notable color deviations compared to the control. 

### 3.4. Sensory Evaluation

**Figure 4:**
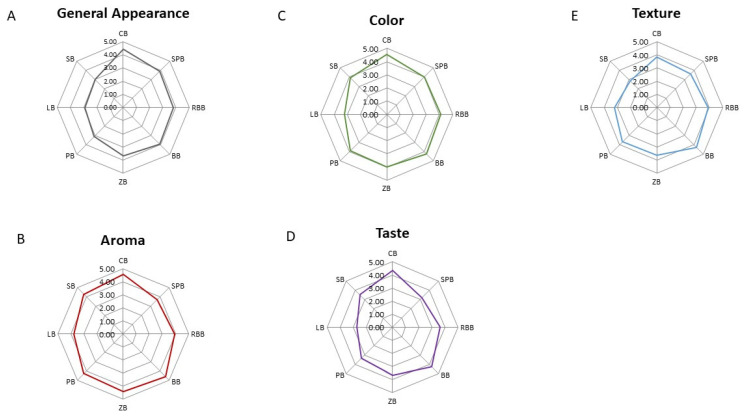
Sensory Evaluation of Brownie Variants: (**A**) General Appearance, (**B**) Aroma, (**C**) Color, (**D**) Taste, and (**E**) Texture. CB—classic brownie, SPB—sweet potato brownie, RBB—red bean brownie, BB—beetroot brownie, ZB—zucchini brownie, PB—pumpkin brownie, LB—lentil brownie, SB—spinach brownie.

The results of the sensory evaluation of brownies are presented in Figure 4A–E, with the control brownie (CB) consistently receiving the highest scores across most sensory attributes. In terms of general appearance (Figure 4A), CB scored the highest (4.45), followed by beetroot brownie (BB) and sweet potato brownie (SPB), with scores of 3.95 and 3.89, respectively. The lowest scores were observed for lentil brownie (LB) and spinach brownie (SB), with LB receiving the lowest score (2.89). For aroma (Figure 4B), BB had the highest score (4.58), followed closely by CB (4.55). Other variants, such as SB and zucchini brownie (ZB), also performed well, with scores of 4.26 and 4.39, respectively. The lowest aroma ratings were noted for SPB (3.71) and LB (3.79), indicating that these variants were perceived as less aromatic by the panelists. The color evaluation (Figure 4C) revealed that CB had the highest score (4.58), indicating that its color was the most preferred. BB and red bean brownie (RBB) also performed well in this category, with scores of 4.24 and 4.11, respectively. The lowest color score was observed for LB (3.21), suggesting a less appealing color in this variant. For taste (Figure 4D), CB again received the highest score (4.37), closely followed by BB (4.24), indicating that these two variants were the most favored in terms of flavor. ZB, RBB, and pumpkin brownie (PB) had moderate taste scores, ranging from 3.37 to 3.66, while LB received the lowest score (2.71), indicating that it was the least preferred in terms of taste. In terms of texture (Figure 4E), BB was rated the highest (4.29), followed by RBB (3.89) and CB (3.82). The lowest texture ratings were assigned to SB (2.87) and LB (3.21), indicating that these samples had a less desirable texture compared to the other brownies.

The overall desirability results for the brownies are summarized in Table 4. The classic brownie (CB) received the highest overall desirability score (8.02 ± 0.46) with a likeability index (LA) of 89.10%, indicating strong panelist preference. Similarly, beetroot brownie (BB) had a high desirability score (7.85 ± 0.52) and an LA of 87.23%, both falling within the range of “like moderately” to “like very much.” Red bean brownie (RBB) and pumpkin brownie (PB) were rated moderately, with desirability scores of 6.96 ± 0.48 and 6.04 ± 0.67, and LA values of 77.32% and 67.16%, respectively. SPB had a desirability score of 5.96 ± 0.66 and an LA of 66.20%, showing slightly lower acceptability, while zucchini brownie (ZB) scored 5.07 ± 0.65 with an LA of 56.30%, falling below the acceptable threshold. Spinach brownie (SB) and lentil brownie (LB) had the lowest desirability scores (5.11 ± 0.44 and 2.94 ± 0.39, respectively), with LA values of 56.82% and 32.69%, indicating low sensory acceptance.

### 3.5. Correlations Between Variables in the Evaluation of Brownies

Table 5 presents the results of the correlation analysis between various physicochemical and sensory attributes of the brownie samples. A moderate positive correlation was observed between moisture and fiber (r = 0.489), suggesting that higher moisture content is associated with increased fiber levels in the samples. In contrast, moisture exhibited a moderate negative correlation with hardness (r = −0.482), indicating that higher moisture content leads to a reduction in the hardness of the brownies. The relationship between moisture and texture was found to be negligible (r = −0.039), showing no significant impact of moisture on texture. Fat content demonstrated a moderate positive correlation with both hardness (r = 0.424) and taste (r = 0.555), suggesting that higher fat levels contribute to increased hardness and improved taste perception. The correlation between fat and texture was weakly positive (r = 0.259), indicating a minimal effect of fat on the textural properties. Similarly, fiber content showed weak positive correlations with hardness (r = 0.202) and texture (r = 0.059), suggesting that fiber has a limited influence on these attributes. In terms of sensory parameters, general appearance showed a very strong positive correlation with color (r = 0.868), indicating that the visual appeal of the brownies is highly dependent on their color. Strong positive correlations were also found between general appearance and both taste (r = 0.767) and texture (r = 0.705), highlighting the importance of these factors in shaping the overall appearance. A weaker positive correlation was noted between general appearance and aroma (r = 0.359), suggesting that aroma plays a less prominent role in the perception of the brownies’ appearance.

## 4. Discussion

According to the World Health Organization: “People are now consuming more foods high in energy, fats, and free sugars or salt/sodium, and many do not eat enough fiber-rich fruits, vegetables, and whole grains” [32]. This is why we decided to propose some modifications to the composition of the brownie, which is a popular and delicious cake. Indeed, expected positive changes in the chemical composition of brownies after butter replacement by selected plant-derived ingredients (sweet potato, red beans, beetroot, zucchini, pumpkin, lentil, and spinach) were observed (see Table 2). Along with the decrease in the fat content, the enhanced amounts of dietary fiber and ash in all cake variants were determined. The most valuable source of dietary fiber has been found to be a variant with sweet potatoes (SPB) (93% higher value than obtained for CB). In turn, the same variant was characterized by the highest values of ash (36% higher than from CB). Regarding ash, its content value was related to mineral content in brownies, and—according to Sumartini et al. [3]—the components present in inorganic compounds are usually potassium, calcium, sodium, iron, magnesium, and manganese.

The replacement of the whole amount of butter with pureed vegetables resulted in a dramatic decrease in the fat content, as seen in all cake variants. These modifications resulted in about 3-fold lower levels of this parameter (Table 2) and a desirable reduction in the energy value of the brownie, a maximum of 57% as compared to the classic cake (Figure 2). Sumartini et al. [3] mentioned important factors for the final results, such as temperature, baking time, and chemical reactions that occur in amino acids with other chemical compounds. In the case of protein levels in brownies, lentil-enriched cake was the most abundant (Table 2). Similarly, as in the case of previously mentioned parameters, the obtained values were influenced by the initial levels of examined parameters in the raw materials added instead of butter. Frequent consumption of products rich in fats and sugars, such as traditional brownies, contributes to the growing prevalence of non-communicable diseases (NCDs), including obesity, diabetes, and cardiovascular diseases. Addressing these risks through dietary modification aligns with the World Health Organization’s recommendation to promote fiber-rich diets, particularly through increased vegetable intake [5]. The modifications introduced in this study not only reduce fat content but also increase dietary fiber and add health-promoting bioactive compounds, such as polyphenols and carotenoids, present in vegetables like sweet potatoes and spinach [8,33]. These nutrients contribute to antioxidant and anti-inflammatory effects, further supporting NCD prevention. By incorporating vegetables into a familiar format, such as brownies, this approach could help overcome common barriers to healthy eating, especially among individuals at high risk for NCDs. Moreover, such modifications could be applied to other baked goods, broadening the scope of functional food development and its impact on public health nutrition.

That means that these positive changes in the proximal composition of brownies were a direct consequence of incorporating raw plant materials containing the valuable compounds into the cake recipe. It is, however, worth noting that due to their presence in the brownies and other health-promoting substances, we have not determined in this study to enrich the final product in vitamins, minerals, and phytochemicals. The main difference is that each vegetable group contains a unique combination and amount of these compounds, which distinguishes them from other groups and each vegetable—within its own group [8]. 

Knowing that, we have prepared seven variants of cake, with a different vegetable raw material. Particular attention should be paid to moisture and dietary fiber. According to Sumartini et al. [3], the moisture of the final product is influenced by several factors. Among them, not only the initial water level of the raw materials and the length of the cake baking but also the amount of dietary fiber in the brownies is to be regarded. Rodrigues et al. [33] reported that dietary fiber may have caused the water absorption index and water retention power to increase. This finding is consistent with those of Okorie and Onyeneke [34] and also Samiha [35], who found that the moisture content of their cake increased significantly after product recipe modification (flour obtained from sweet potato instead of wheat flour). Moreover, the enhanced values of this parameter were observed by Grigelmo-Miguel et al. [36] for muffins with peach fiber, partially replacing the fat used. Strong water-binding properties were confirmed also by Baixauli et al. [37] and Kowalska et al. [38]. The greater moisture content can be linked to the water retention capacity of dietary fiber. In our study, a correlation coefficient of 0.49 was obtained for moisture and dietary fiber, indicating only a medium correlation (Table 5).

According to Kowalska et al. [38], water content, being the highest in beetroot brownie (among all cakes in our study), is one of the main factors that affect the texture of cakes. Indeed, the increased moisture content in brownies after the incorporation of orange sweet potato puree was regarded as the major reason affecting the textural changes in the study of Selvakumaran et al. [39]. These authors stated that even if increased fiber is associated with increased hardness, the enhancement of moisture content with puree addition may overcome the effect of increased fiber in brownies, resulting in reduced hardness with a cake-like texture. These observations were not confirmed by the results of our study, where only a weak correlation was observed for dietary fiber and hardness (0.20) and a medium negative correlation (−0.48) for moisture and hardness (Table 5). However, higher water content could be a positive factor in sensory analysis if this kind of modification caused the product to be perceived as moister [40]. In our study, beetroot brownie obtained the highest score for texture (4.29) (Figure 4). 

The difference in the textural properties was explained by different authors in relation to the cake ingredients. A softening function of margarine, eggs, and milk powder in brownies was reported in various studies by Mardiana et al. [41]. In the classic brownie recipe, only eggs from the above-mentioned ingredients were used, and butter was chosen instead of margarine (Table 1). The next issue to consider could be thermal processing conditions. Setyaningsih et al. [42] proved that the length of time for the thermal treatment is notable due to the water evaporation and fat melting. Thus, the optimization of temperature could be one of the further steps regarding these aspects. It is important to remember that even the best composition of a product will not be a sufficient argument for consuming it instead of the typical, classic version if it is not organoleptically acceptable. In the case of brownies, attributes such as general appearance, aroma, color, taste, and texture are regarded as important. It was shown that in the case of all parameters except for texture and aroma, the classic brownie was the most desirable version (Figure 4). 

Fat influences the texture and sensory characteristics of the food product and, consequently, its acceptability to consumers [43]. Therefore, the replacement of fat is not an easy but achievable task. As an example, the study of Fleischer [7] could be mentioned. The formulations of brownies that included black bean purees as fat replacers made it possible to reduce overall fat content without compromising the sensory properties of the product. 

In the case of our raw materials, the use of vegetables was related to decreasing scores of such organoleptic attributes as general appearance, color, and taste compared to classic brownies. The lowest marks from consumers were obtained for lentil brownies in the case of general appearance parameter (2.89), color (3.21), and taste (2.71). In the case of texture, the lowest marks were obtained for SB and for aroma—SPB (Figure 4). Studies have shown that visual appeal is a critical factor in consumer acceptance of modified baked goods. For instance, previous research indicates that maintaining a familiar appearance is essential when introducing novel ingredients, as deviations can lead to lower acceptability scores [44]. The low score of the spinach brownie (SB) suggests that the green coloration was not well received, aligning with findings by other studies where non-traditional colors in familiar products led to decreased visual appeal [45]. 

The highest score in overall appearance (Figure 4A) was awarded to the classic brownie (CB), which outperformed the modified versions like the zucchini brownie (ZB), beetroot brownie (BB), and red bean brownie (RBB). Studies have shown that visual appeal is a critical factor in consumer acceptance of modified baked goods. For instance, previous research indicates that maintaining a familiar appearance is essential when introducing novel ingredients, as deviations can lead to lower acceptability scores [44]. The low score of the spinach brownie (SB) suggests that the green coloration was not well received, aligning with findings by other studies where non-traditional colors in familiar products led to decreased visual appeal [45]. Aroma plays a crucial role in the sensory perception of food, influencing overall acceptance. The lower scores for beetroot brownie (BB) and spinach brownie (SB) indicate that the vegetable ingredients imparted fewer desirable odors, which could detract from the classic brownie experience (Figure 4B). According to the literature, vegetables with distinct or earthy odors, like beetroot and spinach, can impact the aromatic profile of baked products negatively, as seen in similar sensory evaluations of vegetable-enriched baked goods [46]. The fact that the other modified brownies, including ZB, maintained aroma scores comparable to CB highlights the importance of selecting ingredients that blend harmoniously with traditional dessert aromas.

Color is a primary sensory attribute that affects consumer perception even before tasting. The zucchini brownie (ZB) maintained a color close to the classic brownie (Figure 4C), supporting studies that suggest mild-colored vegetables do not drastically alter the visual properties of baked goods [47]. In contrast, the spinach and sweet potato brownies (SB and SPB) received lower scores, which corresponds with findings in the literature that products deviating too far from expected color profiles often receive lower sensory ratings [48].

According to the LA criteria, a score of 70% or higher is considered acceptable for sensory properties [39,49]. In Table 4, the classic brownie (CB) achieved the highest LA at 89.10%, indicating excellent acceptability, which is well above the 70% threshold. Similarly, brownies enriched with black beans (BB) had an LA of 87.23%, demonstrating high sensory acceptability. Brownies made with red beans (RBB) also met the acceptability criteria with an LA of 77.32%, while other formulations, such as pumpkin brownies (PBs) and sweet potato brownies (SPB), fell below the acceptable threshold, with LAs of 67.16% and 66.20%, respectively. Notably, zucchini brownies (ZB) and lentil brownies (LB) had the lowest LA values, at 56.30% and 32.69%, indicating poor sensory acceptability. These results suggest that not all formulations were equally successful in maintaining desirable sensory properties, with some falling significantly short of the 70% LA threshold required for acceptance.

Taste is the most critical attribute, especially in novel food products. The taste scores reflect that zucchini brownies (ZB) were closest in flavor to the classic brownie (Figure 4D), suggesting that zucchini’s subtle taste integrates well with the sweet, chocolatey profile. Previous studies have also identified zucchini as a favorable vegetable addition to baked goods due to its neutral taste and moisture-retaining properties [47]. Conversely, the lower taste score for spinach brownies (SB) highlights the challenges of incorporating strongly flavored vegetables into desserts without significantly altering the expected taste profile. The “metallic aftertaste” noted in the red bean brownie (RBB) aligns with known issues in sensory evaluations involving legumes, especially when canned products are used [46]. Texture (Figure 4E) showed that ZB was perceived to have the most desirable texture among the modified variants, likely due to the zucchini’s ability to retain moisture without altering the crumb structure. This observation is consistent with other studies that have explored the textural benefits of incorporating vegetables like zucchini into cakes, where they act as natural humectants, improving texture without compromising integrity [44]. The lower texture score for sweet potato-infused brownies (SPB) may be attributed to its starch-heavy composition, which can lead to a denser and less desirable mouthfeel, as reported in similar studies on starch-rich vegetable additions [45]. 

Important information related to the effects of brownie modification on the final product features is also based on the results of the instrumental methods used for texture analyses and color measurements. 

Among the texture parameters, the hardness, chewiness, coherence, and springiness of the brownies were affected by the different formulations used (Figure 4A–D). That means that the addition of vegetables significantly affects the texture of the prepared brownie variants, modifying properties such as hardness, chewiness, coherence, and springiness. Statistical tests revealed significant differences between the classic brownie (CB) and vegetable-enriched variants, as well as among the different variants.

Hardness is a key textural parameter that influences the sensory perception of baked products [50]. No statistically significant differences from CB were observed for sweet potato brownies and red bean brownies in the case of hardness (Figure 4A). In contrast, the beetroot and pumpkin addition led to a decrease in the value of this parameter, suggesting the role of these raw materials in softening the cake texture (*p* < 0.05). ZB, LB, and SB were notably softer, and these differences were statistically significant compared to CB (*p* < 0.05). The observation that brownie variants enriched with zucchini, lentils, or spinach contribute to a softer structure was in line with previous research on vegetable incorporation into baked products [50].

In terms of chewiness, the classic brownie exhibited the lowest value, indicating a crumblier and less chewy structure (Figure 3B). Similar values (*p* > 0.05) were recorded for lentil brownies. Chewiness in baked products is influenced by the gluten network structure and starch content, which contribute to a denser and more resistant texture. The higher chewiness in SPB (as can be seen in Figure 3) may be attributed to the starch content in sweet potatoes, which creates a denser product structure, as confirmed by previous studies [51,52].

For coherence, CB again had the lowest value (Figure 3C) compared to the vegetable variants (*p* < 0.05). This parameter refers to the internal stickiness of the product, and higher values can enhance sensory appeal by giving the product a more cohesive and unified texture. Studies on vegetable-enriched baked goods have shown that plant fibers can increase product coherence by improving water retention and creating a more uniform structure [53].

Springiness, or the product’s ability to return to its original shape after compression, was lowest in CB and LB, indicating their denser texture and lack of elasticity. Sweet potato brownie (SPB) and spinach brownie (SB) exhibited the highest springiness (Figure 3D). This parameter is often associated with the elastic properties of the matrix formed by proteins and starches in the product, and its value is usually enhanced by the presence of plant fibers, as demonstrated in previous studies on fiber-enriched baked goods [54,55].

In turn, the color of the cakes was influenced by the addition of spinach and zucchini, which appear grayish, and by the sweet potato and lentil, which present a yellowish color. From the results obtained, we can conclude that cake variants exhibit significant differences in their color attributes, particularly in terms of lightness, redness, and yellowness (Table 3). The SPB and LB variants were significantly lighter, while BB and PB display the strongest red and yellow tones. ZB and SB, however, stand out due to their darker and more subdued color profiles. The color differences (ΔE) reveal that ZB and SB deviate the most from the other variants, indicating a more noticeable color variation, while RBB and CB maintain the most consistent and less noticeable color variations. 

Taking all the results into account, our study showed that the incorporation of plant-derived raw materials can be successful. The replacement of fat with these ingredients improved the nutritional composition of the brownie as compared to the classic cake made with butter. The new variants of nutritious brownies could provide an interesting way to incorporate portions of vegetables into our diet when delicious dessert is consumed. However, optimization of the recipe to ensure preferable organoleptic attributes of final products is needed.

## 5. Conclusions

This study demonstrates that incorporating plant-derived ingredients into brownie formulations effectively enhances their nutritional profile, notably by reducing fat content and increasing dietary fiber and ash. The substitution of butter with vegetable purees, such as sweet potato, lentils, and spinach, resulted in up to a three-fold reduction in fat and a significant decrease in the caloric content, with the sweet potato brownie (SPB) showing the highest dietary fiber and ash content. However, while these modifications improved the nutritional composition, sensory evaluations indicated that organoleptic properties were impacted. The classic brownie (CB) consistently received the highest scores for appearance, color, and taste, while vegetable-enriched variants, particularly lentil (LB) and spinach brownies (SB), showed lower acceptability in these parameters. Texture analysis revealed that moisture retention, particularly in the zucchini brownie (ZB), improved the textural properties, though correlations between dietary fiber and textural attributes, such as hardness, were weak.

In conclusion, the incorporation of plant-based ingredients in brownies presents a promising strategy to improve the nutritional value of this popular dessert. These vegetable-enriched brownies could offer a novel, health-conscious alternative to traditional brownies, contributing to increased vegetable intake in a familiar format. Additionally, the results of this study have potential applications in both the market and clinical practice. Vegetable-enriched brownies could support dietary interventions aimed at increasing vegetable intake and reducing risk factors for non-communicable diseases (NCDs), such as obesity, diabetes, and cardiovascular diseases, while appealing to health-conscious consumers. These findings suggest that integrating such recipes into public health and dietary strategies could help mitigate the burden of NCDs by providing healthier alternatives to traditional high-fat desserts. However, further optimization is needed to enhance sensory attributes, ensuring consumer acceptance.

## Figures and Tables

**Table 1:** Ingredient List for Classic Brownie and Vegetable-Modified Brownie Variants.

Ingredient [g]	Classic Brownie (CB)	Sweet Potatoes Brownie (SPB)	Red Bean Brownie (RBB)	Beetroot Brownie (BB)	Zucchini Brownie (ZB)	Pumpkin Brownie (PB)	Lentil Brownie (LB)	Spinach Brownie (SB)
Medium-sized chicken eggs	275 (5 psc)	275 (5 psc)	275 (5 psc)	275 (5 psc)	275 (5 psc)	275 (5 psc)	275 (5 psc)	275 (5 psc)
Dark chocolate 70% cocoa	200	200	200	200	200	200	200	200
Wheat flour (type 550)	100	100	100	100	100	100	100	100
White granulated sugar	100	100	100	100	100	100	100	100
Butter, 82% fat	200	-	-	-	-	-	-	-
Plant-based puree	-	200	200	200	200	200	200	200

**Table 2:** Moisture, protein, fat, fiber, ash, and carbohydrate content of the brownie modifications expressed as mean ± standard deviation in g per 100 g.

Cake Variant	Moisture	Protein	Fat	Dietary Fiber	Ash	Total Carbohydrate
CB	20.65 ± 1.30 A	7.48 ± 0.03 A	29.85 ± 0.64 A	4.22 ± 0.32 A	0.92 ± 0.02 A	36.88 ± 0.21 B
SPB	32.95 ± 0.42 Ba	8.01 ± 0.32 Ba	13.30 ± 0.42 1 Ba	8.15 ± 0.05 Bc	1.25 ± 0.05 Bb	36.30 ± 0.10 Bb
RBB	32.11 ± 0.42 Ba	8.26 ± 0.18 Bb	11.00 ± 0.71 Ba	7.95 ± 0.12 Bc	1.23 ± 0.01 Bb	36.30 ± 0.12 Bb
BB	42.22 ± 1.09 Cb	7.69 ± 0.24 Ba	12.10 ± 0.71 Ba	6.35 ± 1.03 Bb	1.14 ± 0.05 Bab	30.50 ± 0.31 Aa
ZB	33.90 ± 0.21 Ba	7.76 ± 0.18 Ba	11.10 ± 0.42 Ba	6.32 ± 1.91 Bb	0.97 ± 0.03 Ba	39.95 ± 0.10 Cc
PB	38.87 ± 0.40 Bb	7.67 ± 0.06 Ba	8.81 ± 0.25 Bb	7.78 ± 0.33 Bc	1.21 ± 0.03 Bb	35.67 ± 0.20 Bb
LB	35.77 ± 0.57 Bb	8.39 ± 0.20 Bb	12.10 ± 0.14 Ba	7.06 ± 0.12 Bc	0.94 ± 0.01 Ba	35.74 ± 0.11 Bb
SB	37.56 ± 0.51 Bb	7.48 ± 0.10 Aa	8.83 ± 0.73 Bb	5.80 ± 0.68 Ba	1.12 ± 0.02 Bab	39.21 ± 0.30 Cc

CB—classic brownie, SPB—sweet potato brownie, RBB—red bean brownie, BB—beetroot brownie, ZB—zucchini brownie, PB—pumpkin brownie, LB—lentil brownie, SB—spinach brownie. Values are presented as means ± standard deviations. A, B, C—different letters denote statistically significant differences between the mean results for the classic brownie and the modified variants (*p* < 0.05); a, b, c—different letters denote statistically significant differences between the modified brownie variants (in dependence on the vegetable additive used) (*p* < 0.05).

**Table 3:** Color parameters (L: lightness, a: redness, b: yellowness, ΔE: total color difference) of classic brownie and its vegetable variants.

Cake Variant	L	a	b	Delta E
CB	26.33 ± 0.7 B	9.88 ± 0.14 C	10.15 ± 0.33 B	0.66 ± 0.33 A
SPB	30.0 ± 2.75 Cc	9.22 ± 0.56 Cc	11.38 ± 1.01 Cc	0.87 ± 0.24 Bb
RBB	25.73 ± 1.18 Bb	9.53 ± 0.28 Cc	14.1 ± 0.55 Dd	0.48 ± 0.41 Aa
BB	25.68 ± 0.64 Bb	10.53 ± 0.08 Dd	14.36 ± 0.15 Dd	0.85 ± 0.65 Bb
ZB	21.98 ± 1.58 Aa	6.21 ± 0.75 Aa	9.61 ± 1.8 Bb	2.37 ± 1.16 Dd
PB	26.32 ± 0.4 Bb	10.0 ± 0.37 Cc	13.84 ± 0.82 Dd	1.03 ± 0.74 Cc
LB	30.2 ± 2.82 Cc	8.77 ± 0.3 Bb	12.12 ± 0.17 Cc	1.97 ± 2.07 Dcd
SB	23.03 ± 1.03 Aa	6.97 ± 0.29 Aa	5.69 ± 0.38 Aa	2.05 ± 1.12 Dd

CB—classic brownie, SPB—sweet potato brownie, RBB—red bean brownie, BB—beetroot brownie, ZB—zucchini brownie, PB—pumpkin brownie, LB—lentil brownie, SB—spinach brownie. Values are presented as means ± standard deviations. A, B, C, D—different letters denote statistically significant differences between the mean results for the classic brownie and the modified variants (*p* < 0.05); a, b, c, d—different letters denote statistically significant differences between the modified brownie variants (i.e., depending on the vegetable additive used) (*p* < 0.05).

**Table 4:** Overall Desirability and Liking Acceptance (LA) Percentage of Brownie Variants.

Cake Variant	Overall Desirability	LA [%]
CB	8.02 ±0.46	89.10
SPB	5.96 ± 0.66	66.20
RBB	6.96 ± 0.48	77.32
BB	7.85 ± 0.52	87.23
ZB	5.07 ± 0.65	56.30
PB	6.04 ± 0.67	67.16
LB	2.94 ± 0.39	32.69
SB	5.11 ± 0.44	56.82

CB—classic brownie, SPB—sweet potato brownie, RBB—red bean brownie, BB—beetroot brownie, ZB—zucchini brownie, PB—pumpkin brownie, LB—lentil brownie, SB—spinach brownie.

**Table 5:** Correlations between variables in the evaluation of brownies.

Compared Variables	Correlation Value (r)
Moisture—Fiber	0.489
Moisture—Hardness	−0.482
Moisture—Texture	−0.039
Fat—Hardness	0.424
Fat—Texture	0.259
Fiber—Hardness	0.202
Fiber—Texture	0.059
Fat—Taste	0.555
General Appearance—Aroma	0.359
General Appearance—Color	0.868
General Appearance—Taste	0.767
General Appearance—Texture	0.705

## Data Availability

The original contributions presented in this study are included in the article. Further inquiries can be directed to the corresponding author.
